# Fast and Reliable Determination of Virgin Olive Oil Quality by Fruit Inspection Using Computer Vision

**DOI:** 10.3390/s18113826

**Published:** 2018-11-08

**Authors:** Javiera Navarro Soto, Silvia Satorres Martínez, Diego Martínez Gila, Juan Gómez Ortega, Javier Gámez García

**Affiliations:** Robotics, Automation and Computer Vision Group, Electronic and Automation Engineering Department, University of Jaen, ES-23071 Jaen, Spain; jpns0002@red.ujaen.es (J.N.S.); dmgila@ujaen.es (D.M.G.); dmmg0001@red.ujaen.es (J.G.O.); jggarcia@ujaen.es (J.G.G.)

**Keywords:** olive, fruit, olive oil, computer vision, olive oil production process

## Abstract

The presence of minor compounds in virgin olive oils has been proven to play multiple positive roles in health protection, encouraging its production. The key factors that influence the oil quality are ripening stages and the state of health of the fruit. For this reason, at the oil mill’s reception yard, fruits are visually inspected and separated according to their external appearance. In this way, the process parameters can be better adjusted to improve the quantity and/or quality of olive oil. This paper presents a proposal to automatically determine the oil quality before being produced from a previous inspection of the incoming fruits. Expert assessment of the fruit conditions guided the image processing. The proposal has been validated through the analysis of 74 batches of olives coming from an oil mill. Best correlation results between the image processing and the analytical data were found in the acidity index, peroxide values, ethyl ester, polyphenols, chlorophylls, and carotenoids.

## 1. Introduction

Due to its healthy composition and great commercial value, the Virgin Olive Oil (VOO) market is in current expansion [1]. Emerging countries are developing their own olive industries, thereby increasing competition among traditional Mediterranean producers [2]. Given this new scenario, widening the range of products is an opportunity to increase profits by opening up new markets.

Existing legislation [3] recognizes only three VOO commercial categories (in the order of highest to lowest quality): Extra Virgin Olive Oil (EVOO), Virgin Olive Oil (VOO), and Lampante Virgin Olive Oil (LVOO). These categories are marked mainly by degradation parameters such as the acidity index, peroxide values, ethyl esters, K232 and K270. The limit values of these parameters are progressively less strict for each of those categories. When the values reach the threshold for Lampante, the oil cannot be consumed and needs to be refined before doing so.

Olive oil is extracted in the oil mill using exclusively mechanical means. That is why the incoming fruits play a major role in the quality of oil. The mechanical extraction allows the presence of minor compounds, granting taste, aroma, and colour. These holistic features are a potential distinction for commercial brands [4].

Minor compounds include two main groups: polyphenols and pigments, which are responsible for relevant health properties and also for increasing the shelf life [5]. Polyphenols are the main group, acting as antioxidant and antiradical agents [6]. These properties have been recognized, in a specific health regulation approved by the European Commission [7], because of its contribution to protecting blood lipids from oxidative damage. In this matter, a recent review [8] proposes to extend the types of polyphenols considered in the EC 2012 claim. The other group, pigments, which are related to oil colour [9], protect against degenerative diseases. Green tones are associated with the chlorophyll content, while yellow tones are related to carotenoid components [10]. Both pigments act as antioxidants or prooxidants, depending on the storage conditions [11].

The quantity of these minor compounds is influenced by the olive variety, the fruit ripeness and the environmental conditions [6]. Considering the whole fruit development, the polyphenol content increases with the degree of ripeness until it reaches a maximum at the fully ripe stage. Afterwards, the quantity of polyphenol decreases in overripe stages. Consequently, chlorophyll and carotenoids show a similar behaviour. Both reach a maximum value at the unripe stage, decreasing as fruits ripen and almost disappearing at the overripe stage. In addition, the carotenoids loss is less severe than that of chlorophyll [12].

Overripe fruits have purple or black skin colour and show a lack of turgor. At this stage, they are vulnerable to harvest damage and wind falls with the subsequent external ground contamination, increasing the risk of spoiling the oil. Overripe stages are not strictly related to low quality oil, although a careful manipulation is required to avoid any fruit damage at harvest and storage [13].

The ripe stage of the fruits and their quality are manually assessed at the reception yard. The objective of this inspection is twofold. First, the production of similar oil lots requires a homogeneous incoming fruits. Second, damaged fruits should be rejected or stored separately for further processing. The fruit assessment is normally done by a trained operator. This task is subjective, time-consuming, highly influenced by the lighting conditions, and subject to errors.

For those reasons, it is highly recommended to automate this procedure. Computer vision has been proven as a feasible technology to couple external fruit inspection with accurate results [14]. Riquelme et al. [15] identified different types of olive skin damage, mainly at the unripe stage, using colour and texture features. Further research done by Ram et al. [16] incorporated shape, colour and texture features to determine the fruit oil content. It was successfully applied to sets of ten fruits of just one variety. Furferi et al. [17] established a correlation among the fruit ripeness, obtained by computer vision, and the content of polyphenol, oil and sugar in fruit from different varieties. Guzmán et al. [18] established different levels of external damage in fruit lots acquired by an infrared sensor. In Cáceres et al. [19], the oil acidity index and peroxide values were accurately correlated with colour and texture features by an Artificial Neural Network. A separation of fruits coming from the ground and trees, in three different varieties, was achieved by processing the skin texture in [20].

In the same context, an estimation of the olive-fruit mass and its size was done through the analysis of correlations between fruit images and reference weight measurements in [21]. Other non-invasive technologies such as electronic noses [22] and hyperspectral imaging [23] have been applied to food inspection or remote sensing applications for monitoring environmental resources [24,25]. Computer vision techniques have also been applied to measuring the main variables of olive tree architecture [26] and for oil spill surveillance [27]. Recently, the research group Benalia et al. [28] contemplated the automatic classification of one to one olives, according to a standard index [29]. Despite the laborious task, principal component analysis at the CIELAB colour space was successfully performed for class determination. Best results were found at green stages.

The aim of this study is to determine olive oil quality parameters by processing images acquired from olive fruits. The image processing is based on expert knowledge. More specifically, an exhaustive evaluation of the fruit condition, made by an expert, has been done and correlated with analytical data of minor compounds and degradative parameters. This valuable information has been included in the image processing to determine, in a fast and reliable way, the oil quality. The results of this work will be of great interest for olive oil producers. Having an estimation of the oil quality before its production will enable to improve the adjustment of the production parameters.

The rest of the paper is organized as follows. Section 2 details the proposed methodology presenting the results of the visual assessment, the hardware set-up for the image acquisition and the image processing algorithms. Section 3 shows the experimental validation of the proposal and a discussion of the results. Finally, the conclusions are drawn in Section 4.

## 2. Material and Methods

**Figure 1:**
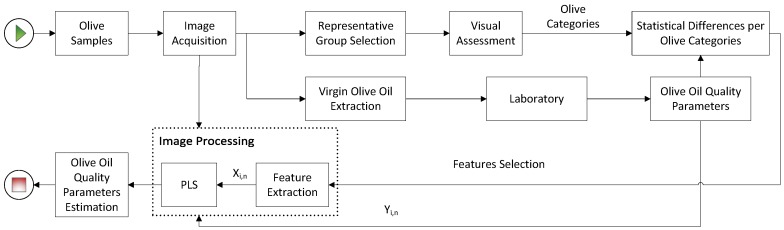
Overview of the proposed methodology for quality properties determination.

To evaluate the link between the olive fruit appearance and the quality of its extracted oil, the methodology shown in Figure 1 was applied. Different olive fruit batches were sampled from various producers when they arrived at the olive oil mill. Images from these batches were acquired and a representative group of olives was selected for each batch to visually assess their physical appearance. After this, all the olive batches were processed into oil, and this oil was chemically analysed. Statistical comparisons were performed between the physical appearance of the fruits and the quality parameters of the oil. This comparison enabled us to establish significant relationships between the human visual perception and the quality parameters obtained by analytical methods. Hence, the criteria used in the visual inspection were employed to select and extract features from the acquired images. These features served as a basis for the development of predictive models, based on PLS regressions, to automatically achieve our goal, the determination of olive oil quality parameters.

### 2.1. Olive Batches Preparation and Oil Extraction

From the end of November 2017 until January 2018, a total of 84 olive batches were obtained weekly from an Olive Oil Cooperative and all of them were used by the expert in the visual assessment. The batches were randomly collected after the cleaning step and before the hopper storage at the mill yard. Each batch consisted of 3 kg of olives and they were shipped to the Group of Robotics, Automation and Computer Vision (GRAV) facilities at the Universidad of Jaén with a view to being processed in less than 12 h. A representative group of 100 fruits were selected for assessing the ripeness and the health state of the batch. Additionally, two groups of approximately 250 g. were used to acquire the corresponding images and then they were returned into their respective batch. Finally, each olive batch was processed to extract its corresponding virgin olive oil (VOO).

For the VOO extraction task, an industrial extraction factory system at a laboratory scale was used. The registered name is Abencor and it consisted of a hammer mill, a thermomixer and a centrifugal machine. Each batch was processed with the same process variables, that is, the olive paste was kneaded for 40 min at $30{\;}^{{}^{\circ}}$C and it was centrifuged at 3000 rpm for 90 s. The extracted oil was disposed into dark glass 125 mL bottles (from now on defined as samples). Samples were identified by its batch number and delivered to the accredited chemical laboratory for its analysis.

The chemical analyses were performed three times following standard methodologies in the accredited laboratory CM Europa S.L. The acidity index, peroxide values, K270 and K232 were analysed according to the EU REG. 2568/91. Ethyl esters were obtained following the EU REG. 2569/91. For polyphenols the COI/T.20/Doc No 29 was used. Finally, pigments were measured by spectrophotometry according to Minguez-Mosquera et al. [9].

### 2.2. Visual Assessment

According to the fruit colour and turgor, three ripening groups were initially established. Thus, batches containing unripe fruits with a higher green component were catalogued in the first group, including those ones with totally green or with at least more than 50% of green colour in their skin. The second group consisted of ripe batches with more than 50% of the fruits with purple and black colours, including those completely dark but sustaining the turgor of the fruit. Finally, the third group was assigned to overripe batches containing fruits with completely dark colours, but still tender. This methodology does not include the official eight ripe stages (COI/OH/Doc. No 1, 2011), because some indexes can only be evaluated by peeling the fruit which is not applicable to a non-invasive system; as described in our proposal.

**Figure 2:**
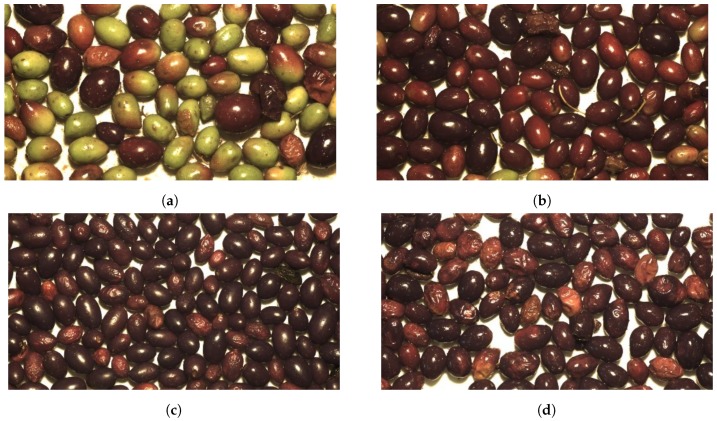
Fruit categories defined by the visual assessment. (**a**) Category 1. Most of the fruits are totally green or with more than 50% of green colour. (**b**) Category 2. Purple or black fruits with an optimal health condition. (**c**) Category 3. Purple or black fruits including spoiled fruits. (**d**) Category 4. Overripe fruits including tender fruits.

Each batch was also evaluated by its general appearance, regarding its health, cleanness or damage condition, which could influence the final oil quality. After these evaluations, the aforementioned second group was subdivided into two groups. The first group involved batches with a higher percentage of ripe fruits and with optimal health conditions. The second group included batches with a high number of ripe but, in this case, spoiled fruits. Therefore, considering the ripeness of the fruit and its external appearance, the four categories shown in Figure 2 were defined.

The olive oil quality parameters for the former categories are presented in Table 1. It is shown that the acidity index was not influenced so much by maturity stages as it was by the fruit health. This issue is revealed by the significant differences observed between *Cat-2* and *Cat-3*. A similar tendency was also followed by the peroxide index. On the contrary, the ultraviolet absorbance parameter K232, which indicates primary oxidation metabolites, was significantly different between *Cat-1* and the others. Due to the quick processing for oil extraction, the parameter K270, which indicates secondary oxidation products, did not give a significant variation among categories. Provided that a new analysis is performed after a period of storage, this parameter could show substantial differences. The last parameter related to the degradation process of the fruits, ethyl esters, had a major impact depending on the fruit health condition.

In case of polyphenols, however, their contents were higher in *Cat-2* with a tendency to decrease in *Cat-3*, according to fruit poor health conditions. Also, significant differences were found between *Cat-1* and *Cat-2* because these compounds were still being generated on the fruits from unripe to ripe stages.

As for the chlorophyll and carotenoid pigments, the first two categories presented higher levels of these compounds. The maturing process involves losses of chlorophyll and carotenoid compounds. Changes start in the skin and then grow into the pulp. So, even in the early black fruit stages, compounds of chlorophylls and carotenoids still remain.

These statistical results were useful to justify the extraction of colour and texture features from the acquired images in order to develop predictive models to estimate the aforementioned olive oil chemical parameters.

### 2.3. Image Processing

The olive fruit images for further processing were acquired using an ad-hoc hardware set-up. In the acquisition set-up, olive batches were placed into a methacrylate rectangular tray (250×165×20 mm) with a white background. Images were acquired by means of a CMOS camera MAKO G-223C with colour sensor, 2048 × 1088 resolution and 5.5μm pixel size. The camera with a lens of 25 mm was positioned at 600 mm. With the former conditions the camera field of view was 270×143 mm, enabling the image acquisition of a large part of the tray.

The lighting system consisted of one 125 W halogen lamp placed in the camera optical axis. This high luminance lighting system allows the camera to run at its maximum frame rate, 50 fps. Due to technical problems with the lighting system, batches corresponding to 21st of November were discarded. Hence, a total of 74 batches were used in the image processing.

Once acquired, the images were preprocessed to remove the background and different features, extracted from the resultant images. These features were correlated with the chemical parameters to obtain the predictive models. Both issues are detailed in the next subsections.

#### 2.3.1. Features Extraction

**Figure 3:**
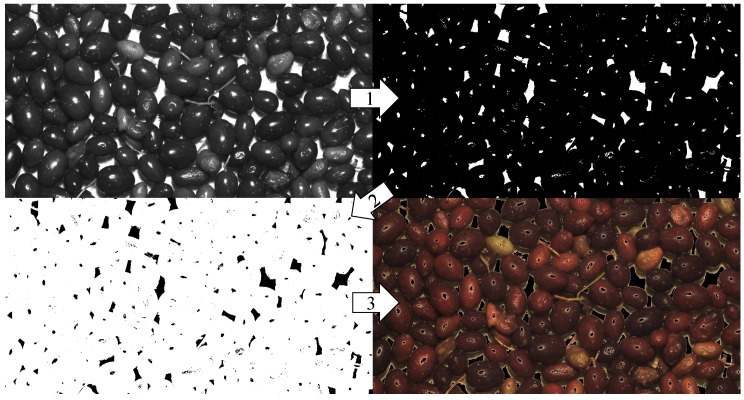
Image processing for features extraction.

The main goal of this step was to extract the useful information from the acquired images. First, the original images in the RGB colour space ($A_{r,g,b}$) were turned into grey level images and then binarized. To extract olives from the white background, a global threshold binarization algorithm was used and the threshold was heuristically fixed. The inverse of the resultant logical mask ($\bar{M}$) was applied to the original images through the logical AND operator (Equation (1)). The result for each step can be seen in Figure 3.

(1) $$I_{r,g,b}=A_{r,g,b}\times \bar{M}$$

Now, colour and texture features can be extracted from the images. The masked RGB images $I_{r,g,b}$ were turned into HSV ($I_{h,s,v})$ and Lab ($I_{l,a,b}$) colour spaces. Then, the average of the grey levels for each channel was computed by (Equation (2)).

(2) $$\bar{I_{c}}=\frac{\sum_{n=1}^{N}\sum_{m=1}^{M}I_{c}\left(n,m\right)}{N\times M}$$

where $\bar{I_{c}}$ is the mean intensity of the different *c* channels and N×M is the number of pixels in the image.

The texture features were extracted from the images according to the Haralick descriptors [30]: angular second moment, contrast, correlation, variance, inverse difference moment, sum average, sum variance, sum entropy, entropy, difference variance, difference entropy, information measures of correlation and maximal correlation coefficient. The former features, along with the colour ones, were used to build the matrix of features $X_{i,j}$ where each row *i* belongs to the batch and the columns *j* are the extracted features from the batch (a total of 23 features). Finally, different vectors $Y_{i,n}$ were coded for each *n* analytical result.

#### 2.3.2. Regression Model

The regression model, based on Partial Least Squares (PLS) [31], was applied to correlate the image features with the analytical reference values. PLS models try to find the multidimensional direction in the $X_{i,j}$ space that explains the maximum multidimensional variance direction in the $Y_{i,n}$ space. The general model can be explained according to Equations (3) and (4).

(3) $$X_{i,j}=T_{i,lv}\cdot P_{j,lv}^{T}+E_{i,j}$$

(4) $$Y_{i,n}=U_{i,lv}\cdot Q_{n,lv}^{T}+F_{i,n}$$

where *T* and *U* are the score matrices, *P* and *Q* are the loading matrices and *E* and *F* are the error terms, all of them of *X* and *Y*, respectively. The number of selected latent values is denoted by *lv*.

**Figure 4:**
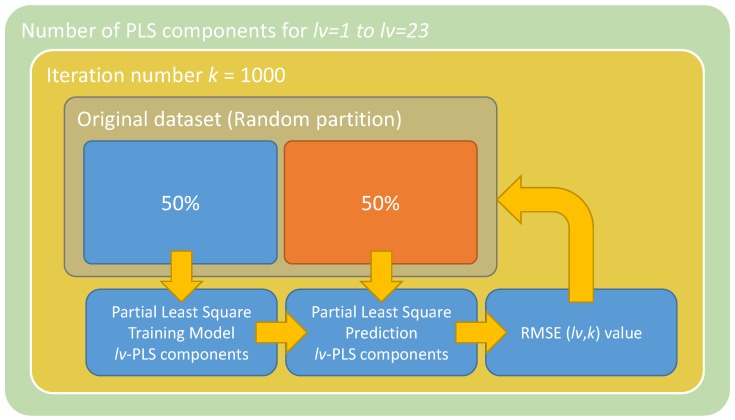
Diagram of the proposal methodology to develop and validate the PLS regression models.

The PLS algorithm was applied to each analytical parameter through the methodology presented in Figure 4. It was an iterative process, where the number of latent values used by the model was increased from 1 to 23 according to the maximum number of latent values, which depended on the number of features. For each iteration, the model was trained and validated 1000 times by using the 50\50 holdout validation process. The prediction ability of the models was compared by means of the following parameters: Root Mean Square Error (RMSE) (Equation (5)), the Regression coefficient ($R^{2}$) (Equation (6)) and the Ratio of Performance to Deviation (RPD) (Equation (7)).

(5) $$RMSE=\sqrt{\frac{\sum_{i=1}^{N}{\left(y_{i}-\hat{y_{l}}\right)}^{2}}{N}}$$

(6) $$R^{2}=1-\frac{\sum_{i=1}^{N}{\left(y_{i}-\hat{y_{l}}\right)}^{2}}{\sum_{i=1}^{N}{\left(y_{i}-\bar{y}\right)}^{2}}$$

(7) $$RPD=\frac{\sqrt{\frac{1}{N-1}\cdot \sum_{i=1}^{N}{\left(y_{i}-\bar{y}\right)}^{2}}}{RMSE}$$

where *N* is the number of predicted samples, $y_{i}$ is the target value, $\hat{y_{i}}$ the predicted value and $\bar{y}$ is the average value of the analytical reference value.

## 3. Results and Discussion

This section introduces and discusses the different results achieved in this paper. First, the characterization of the olive batches, used in this research, is presented through the analytical parameters of the oil samples. This will serve as a basis for a better understanding of the data distribution for the analytical parameters shown in the second part of this section, the image processing results.

### 3.1. Laboratory Results for the Analytical Parameters

With the purpose of establishing the oil quality, the most outstanding parameters were analysed in each oil sample. Table 2 shows the maximum, minimum, mean and standard deviation for the quality parameters in eight sampling dates. The number of olive batches, obtained to produce the oil samples, varied according to the production capacity of the oil mill. Fruits for each batch were randomly chosen from the conveyor belts, located in the reception yard. The random selection of batches entailed different producers and locations, which implied differences in ripening stages and health conditions. Values for the standard deviation in the analytical parameters show that the lack of homogeneity in the batches was not related to the date.

Additional information can be obtained from Table 2. In general, the acidity index, peroxide values, K270, K232, ethyl esters, including ethyl palmitate and ethyl oleate tend to rise in the last sampling dates. This is due to a prevalence of overripe fruits at the end of the season. If we had extended the period of time in this study (after January) more samples with higher values of these components would have been obtained.

On the contrary, polyphenols, chlorophyll and carotenoids follow a usual decrease, as these quality components are present in higher amounts at early ripe stages (mostly present at the beginning of the season). In this case, an early start of the present research would have shown an increase of these parameters.

### 3.2. Image Processing Results

As detailed in Section 2, the olive fruits were visually assessed and correlated with VOO quality parameters (Table 1). This study showed conclusively that the colour and state conditions of the fruits provide a valuable information to determine the quality parameters.

The former knowledge was used to design a methodology, based on computer vision, to automate the expert task. So, the features assessed in the manual process were obtained from the images. In this sense, the maturity of the fruits was analysed by processing images in different colour spaces, and the health state was obtained by computing texture features of the fruit skin. Then, a prediction model based on PLS was developed for each of the chemical parameters related to the VOO quality. The validation results of these models are presented in Table 3.

As shown in this table, every PLS model had a different design since the number of latent variables was different. Most of them presented a regression coefficient above 0.7, despite the fact that most of the fruits were in the ripe and overripe stages. This is a significant advantage compared to early studies in which it was difficult to evaluate these stages [15,28].

**Figure 5:**
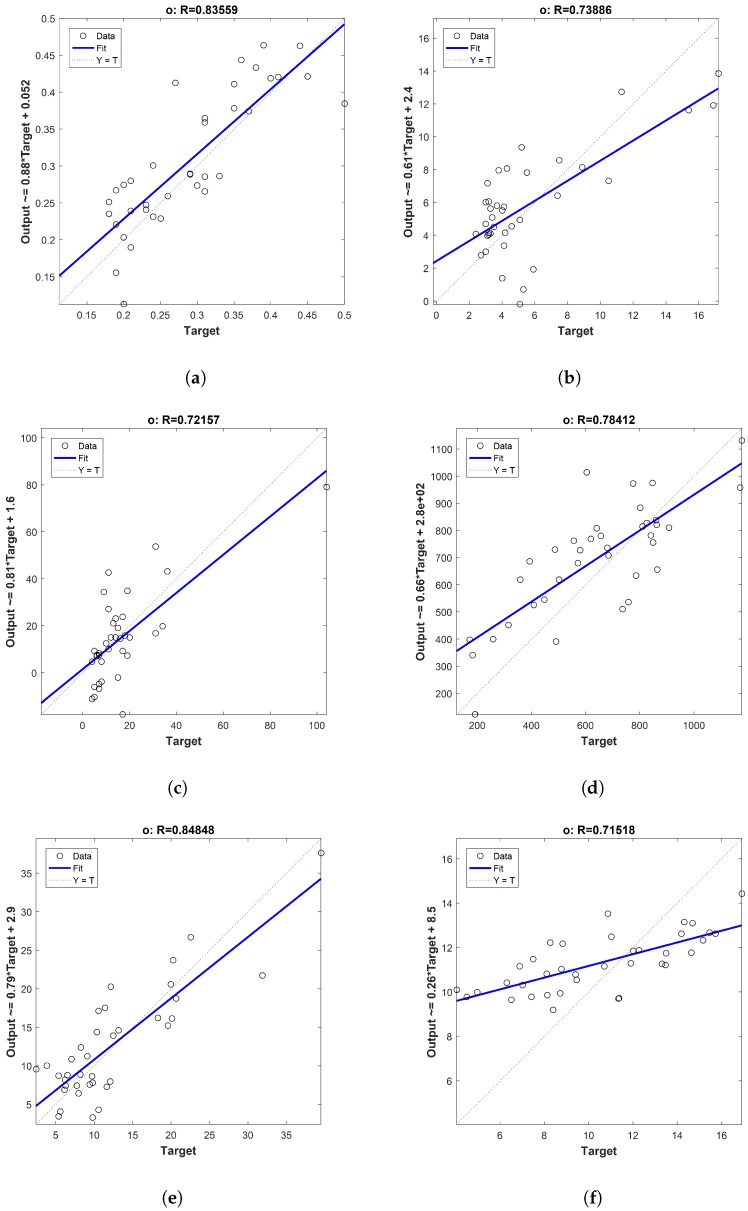
Fruit Image correlations by PLS are presented for different oil parameters. (**a**) Acidity Index. (**b**) Peroxide Index. (**c**) Ethyl Ester. (**d**) Polyphenols. (**e**) Chlorophylls. (**f**) Carotenoids.

The outstanding regression plots are presented in Figure 5. In every sub figure, the *x*-axis is the target (analytical values from chemical laboratory) and the *y*-axis is the regression model output. The best correlation result was for the acidity index Figure 5a. It was a regression coefficient of 0.84, a $RMSE_{v}$ of 0.12 and a RPD of 2.16. As the acidity index is the most important parameter to establish the commercial category of the olive oil, the former values are particularly relevant. Generally, this parameter remains stable throughout the production process and at the oil storage [32], in contrast to the rest of the chemical parameters considered in this study.

Figure 5b corresponds to the peroxide index which also had fine correlation results ($R_{v}=0.74$ and $RMSE_{v}=4.12$). According to [33], this can be easily explained, since this parameter rises when the olive fruit is spoiled and it represents the primary oxidation compounds. It is directly proportional to degradation conditions of the fruits, decreasing when they are transformed into secondary oxidation compounds. Then, the secondary oxidation compounds, which were assessed by the K270 parameters, reached lower correlation results ($R_{v}=0.64$ and $RMSE_{v}=0.04$). It means that for this parameter there are no differences among the olive fruit categories, although these differences might appear in the produced oil during its storage. Since K232 is another way of measuring primary oxidation compounds, the correlation results ($R_{v}=0.69$ and $RMSE_{v}=0.15$) were slightly below the peroxide value. However, the assessment of this quality parameter is optional, so this lower fitting is not so critical. In ref. [34], the authors reached a similar conclusion.

Ethyl esters, another degradative parameters which are related with early fermentative reactions, increases in damaged fruits. These parameters are subject of the latest discussion about reducing their limits for best quality oils. Recent research has found that the ethyl ester production starts when the fruits are on the tree and increases at overripe stages [13]. It also has demonstrated that ethyl esters depend on the ripe fruit conditions and varietal influence. This last factor was not considered in this research. So, the correlation results which were $R_{v}=0.72$ and $RMSE_{v}=16.89$ (Figure 5c) presented an accurate fitting.

For the polyphenols content, the fitting presented in Figure 5d is remarkable. This parameter is influenced by many factors such as the variety of fruits, environmental conditions and agronomical practices [6]. High accuracies of the prediction models for this parameter would indicate the potential to improve the health benefits and the shelf life of the future extracted oil. Even though the $RMSE_{v}$ for this parameter is quite high, considered the wide range of it should be taken into account, from 171 to 1275 mg/kg, over the samples (Table 2).

It is important to denote that differences of fitting have been found for pigments. Particularly, chlorophyll (Figure 5e) had the best correlation overall the studied parameters ($R_{v}=0.85$ and $RMSE_{v}=6.71$). This shows that the colour features were properly selected, as this pigment is highly colour skin dependent as confirmed [10]. Conversely, for carotenoids (Figure 5f), the correlation fit was high but not so accurate ($R_{v}=0.72$ and $RMSE_{v}=3.40$). This issue can be explained by the inherent behaviour of these compounds. Their declining is less pronounced and they are more stable among different ripe stages [11].

## 4. Conclusions

The assessment of the olive fruit conditions at the start of the virgin olive oil production process is an issue of critical importance to optimize the quality of the produced oil. Nowadays, it is the master miller who supervises the fruits brought by the farmers and selects the production line according to the quality. In this paper we present the relationship between colour and texture features, extracted from olive fruit images, and different chemical parameters of the olive oil produced from these fruits. To fulfil this task, 10 prediction models have been implemented based on Partial Least Squares (PLS), one for each quality parameter. The best results in the prediction were achieved for the acidity index ($R_{v}=0.84$ and $RMSE_{v}=0.12$) and chlorophylls ($R_{v}=0.85$ and $RMSE_{v}=6.71$). The proposed method could be implemented on-line in an olive mill in order to classify olive batches at the beginning of the industrial process, thus avoiding losses in quality in the produced olive oil. Last but not least, the prediction models integrated into an on-line system could be useful to comply with the regulation RES-2/94-V/06 related to the quality management guides for the olive oil industry proposed by the International Olive Oil Council.

## Figures and Tables

**Table 1:** Minimum, maximum and mean values for each chemical parameter. Statistical differences (Tuckey’s, p≤0.05) are denoted by different letters in the mean column.

		Acidity Index (%)	Peroxide Index (meq/Kg)	K270	K232	Ethyl Ester (mg/Kg)	Ethyl Palmitate (mg/Kg)	Ethyl Oleate (mg/Kg)	Polyphenols	Chlorophyll	Carotenoids
**Cat-1**	Min.	0.17	2.70	0.09	1.26	3.00	2.00	1.00	241.00	9.80	8.83
	Max.	0.25	4.80	0.17	1.51	10.00	4.00	6.00	841.00	34.73	16.53
	Mean	0.20 a	3.87 a	0.13 a	1.38 a	5.77 a	2.92 a	2.85 a	538.31 a	21.26 c	13.20 b
**Cat-2**	Min.	0.12	2.10	0.12	1.34	4.00	2.00	1.00	452.00	6.24	7.49
	Max.	0.31	5.80	0.28	1.92	9.00	4.00	5.00	1275.00	31.88	20.46
	Mean	0.22 a	3.75 a	0.17 a	1.53 b	5.77 a	2.62 a	3.15 a	765.92 b	17.42 bc	12.20 ab
**Cat-3**	Min.	0.17	2.60	0.11	1.39	5.00	2.00	3.00	182.00	6.30	4.96
	Max.	0.59	15.40	0.21	1.70	64.00	15.00	50.00	864.00	20.30	16.91
	Mean	0.35 b	6.76 ab	0.15 a	1.52 b	17.00 ab	4.92 ab	12.00 ab	585.54 ab	12.89 ab	10.50 ab
**Cat-4**	Min.	0.15	2.70	0.10	1.31	5.00	2.00	2.00	171.00	2.42	3.04
	Max.	0.79	22.50	0.20	1.77	104.00	21.00	83.00	846.00	22.90	14.78
	Mean	0.41 b	8.98 b	0.15 a	1.50 b	31.00 b	7.38 b	23.31 b	457.62 a	10.42 a	9.39 a

**Table 2:** Laboratory results for the analytical parameters. The number of samples per day is indicated by “*n*”.

Parameters		Sampling Dates							
		21/11/2017 n=10	28/11/2017 n=10	4/12/2017 n=10	12/12/2017 n=10	18/12/2017 n=11	10/1/2018 n=13	17/1/2018 n=7	23/1/2018 n=13
$\mathrm{Acidity}\;\mathrm{Index}{\;}^{***}$	**min**	0.12	0.17	0.20	0.15	0.19	0.27	0.29	0.15
	**max**	0.25	0.24	0.31	0.30	0.48	0.79	0.50	0.66
	**mean**	0.19	0.20	0.25	0.22	0.28	0.45	0.39	0.40
	**SD**	0.03	0.02	0.04	0.04	0.08	0.18	0.08	0.14
$\mathrm{Peroxide}\;\mathrm{Value}{\;}^{**}$	**min**	2.10	2.80	2.70	2.40	5.10	3.00	2.80	2.60
	**max**	5.80	4.60	4.30	5.40	11.30	17.20	10.90	22.50
	**mean**	4.20	3.58	3.62	3.55	7.43	8.26	4.89	7.55
	**SD**	0.93	0.55	0.56	1.01	1.97	5.50	2.99	6.06
K270	**min**	0.12	0.11	0.09	0.10	0.11	0.12	0.10	0.13
	**max**	0.19	0.28	0.22	0.24	0.23	0.19	0.21	0.21
	**mean**	0.14	0.17	0.16	0.17	0.15	0.15	0.16	0.16
	**SD**	0.02	0.04	0.04	0.05	0.04	0.03	0.04	0.02
K232	**min**	1.29	1.26	1.27	1.31	1.43	1.28	1.31	1.41
	**max**	1.67	1.92	1.76	1.97	1.84	1.68	1.70	1.76
	**mean**	1.41	1.50	1.49	1.63	1.57	1.47	1.56	1.53
	**SD**	0.11	0.17	0.14	0.23	0.14	0.12	0.13	0.11
$\mathrm{Ethyl}\;\mathrm{Esters}{\;}^{*}$	**min**	3.00	5.00	4.00	11.00	5.00	11.00	5.00	8.00
	**max**	7.00	10.00	6.00	34.00	15.00	104.00	57.00	70.00
	**mean**	5.40	7.10	4.90	19.00	9.09	31.77	14.57	24.46
	**SD**	1.51	1.60	0.88	7.42	3.21	27.75	18.82	15.89
$\mathrm{Ethyl}\;\mathrm{Palminate}{\;}^{*}$	**min**	2.00	2.00	2.00	3.00	2.00	3.00	2.00	3.00
	**max**	4.00	4.00	3.00	10.00	5.00	21.00	13.00	15.00
	**mean**	2.07	3.40	2.10	6.30	3.36	7.54	4.14	6.23
	**SD**	0.82	0.70	0.32	2.00	0.92	5.46	3.98	3.14
$\mathrm{Ethyl}\;\mathrm{Oleate}{\;}^{*}$	**min**	1.00	3.00	2.00	6.00	3.00	8.00	2.00	5.00
	**max**	5.00	6.00	4.00	24.00	10.00	83.00	44.00	55.00
	**mean**	2.60	3.80	2.80	12.20	5.82	24.23	10.43	18.15
	**SD**	1.35	1.14	0.79	5.98	2.23	22.45	14.93	12.78
$\mathrm{Polyphenols}{\;}^{*}$	**min**	321.00	329.00	241.00	290.00	358.00	182.00	171.00	221.00
	**max**	782.00	1275.00	1089.00	1176.00	916.00	864.00	861.00	757.00
	**mean**	532.60	739.20	744.60	719.20	662.45	516.54	597.00	535.69
	**SD**	136.45	246.97	232.71	315.84	197.70	239.18	233.79	148.25
$\mathrm{Chlorophyll}{\;}^{*}$	**min**	9.84	8.83	7.49	6.88	9.40	4.96	3.04	4.49
	**max**	17.22	20.46	14.69	22.26	19.41	15.74	7.02	14.78
	**mean**	13.92	13.38	10.54	11.73	13.09	10.47	5.74	10.11
	**SD**	2.33	3.20	2.45	4.58	2.91	3.47	1.56	3.07
$\mathrm{Carotenoids}{\;}^{*}$	**min**	13.51	12.13	6.24	5.40	5.60	2.40	3.20	2.42
	**max**	34.73	31.88	19.58	39.50	19.20	28.60	12.10	14.01
	**mean**	23.80	20.89	11.76	12.80	11.78	15.71	7.50	8.63
	**SD**	6.57	5.25	4.21	10.06	4.69	8.31	3.25	3.20

*: $\frac{mg}{Kg}$; **: $\frac{mEq\;O_{2}}{Kg}$; ***: % Oleic Acid.

**Table 3:** Regression coefficient (calibration and validation), root mean square error (calibration and validation), number of PLS components employed in the models for each chemical parameter and ratio of performance to deviation.

Parameter	$R_{c}^{2}$	$R_{v}^{2}$	${\mathrm{RMSE}}_{c}$	${\mathrm{RMSE}}_{v}$	lv	RPD
Acidity Index	0.89	0.84	0.11	0.12	2	2.16
Peroxide value	0.87	0.74	3.97	4.12	1	1.01
K270	0.83	0.64	0.03	0.04	3	1.28
K232	0.83	0.69	0.13	0.15	4	1.29
Ethyl Ester	0.88	0.72	15.74	16.89	2	1.07
Ethyl Palmitate	0.85	0.68	3.21	3.42	2	1.17
Ethyl Oleate	0.88	0.74	12.67	13.52	2	1.07
Polyphenols	0.88	0.78	200.6	243.50	5	1.32
Chlorophyll	0.91	0.85	5.96	6.71	2	1.08
Carotenoids	0.88	0.72	3.02	3.40	2	1.44

## Abbreviations

The following abbreviations are used in this manuscript:

VOO	Virgin olive oil
VOOs	Virgin olive oils
EVOO	Extra virgin olive oil
LVOO	Lampante virgin olive oil
PLS	Partial least squares
RMSE	Root mean square error
R	Regression coefficient
RPD	Ratio of performance to deviation
